# Ocular *Chlamydia trachomatis* infection, anti-Pgp3 antibodies and conjunctival scarring in Vanuatu and Tarawa, Kiribati before antibiotic treatment for trachoma

**DOI:** 10.1016/j.jinf.2020.01.015

**Published:** 2020-04

**Authors:** Robert Butcher, Becca Handley, Mackline Garae, Raebwebwe Taoaba, Harry Pickering, Annie Bong, Oliver Sokana, Matthew J Burton, Nuno Sepúlveda, Ana Cama, Richard Le Mesurier, Anthony W. Solomon, David Mabey, Fasihah Taleo, Rabebe Tekeraoi, Chrissy h Roberts

**Affiliations:** a Clinical Research Department, London School of Hygiene & Tropical Medicine, Keppel Street, London WC1E 7HT, United Kingdom; b Department of Neglected Tropical Diseases, Vanuatu Ministry of Health and Medical Services, Port Vila, Vanuatu; c Eye Department, Kiribati Ministry of Health and Medical Services, Bikenibeu, Tarawa, Kiribati; d Eye Department, Vanuatu Ministry of Health and Medical Services, Port Vila, Vanuatu; e Eye Department, Solomon Islands Ministry of Health and Medical Services, Honiara, Solomon Islands; f Department of Infection Biology, London School of Hygiene & Tropical Medicine & Centre of Statistics and Its Applications, University of Lisbon, Portugal; g Fred Hollows Foundation, Melbourne, Australia

**Keywords:** Trachoma, *Chlamydia trachomatis*, Anti-Pgp3 antibodies, Vanuatu, Kiribati, Neglected tropical diseases

## Abstract

**Introduction:**

In the peri-elimination setting, the positive predictive value of trachomatous inflammation–follicular (TF), the primary marker used to determine need for antibiotics for trachoma, is suboptimal. Here, three non-TF measures are used to compare two regions where TF prevalence exceeds the threshold for intervention, but where the *Chlamydia trachomatis* (*Ct*) prevalence is different.

**Methods:**

Population prevalence of trachoma was measured in Vanuatu (*n* = 3470) and Kiribati (*n* = 2922). Dried blood spots (DBS) and conjunctival photographs were collected from every survey participant, and conjunctival swabs were collected from those aged 1–9 years. Individuals were tested for blood anti-Pgp3 antibodies, *Ct* DNA at the conjunctiva and severity of conjunctival scarring.

**Results:**

The prevalence of TF in 1–9-year-olds was 16.5% in Vanuatu and 38.2% in Tarawa. 7% of people aged ≥1 year in Vanuatu had conjunctival scarring compared to 27% in Tarawa. The prevalence of ocular *Ct* infection in 1–9-year-olds was 1.5% in Vanuatu and 27.4% in Tarawa. The seroconversion rate amongst 1–9-year-old children in Vanuatu and Tarawa was 0.018 and 0.197 events per child per year, respectively.

**Conclusions:**

Comparing Vanuatu to Tarawa demonstrates several markers that could be used to differentiate the trachoma status of populations in these (and other) locations.

## Highlights

• In Vanuatu, ocular *Chlamydia* infection prevalence is low; in Kiribati it is high.
• In Vanuatu, Pgp3 seroprevalence does not increase in childhood; in Kiribati it does.
• Conjunctival scarring is more common in adults in Kiribati than in Vanuatu.
• Trachomatous inflammation—follicular lacks specificity for ocular *Chlamydia* infection.
• Non-TF markers may help to determine need for interventions against active trachoma.

## Introduction

Trachoma is caused by *Chlamydia trachomatis* (*Ct*). It is the leading infectious cause of blindness worldwide and the target of global elimination efforts.^1^^,^^2^ Trachoma is treated with **s**urgery for those with trachomatous trichiasis (TT), mass drug administration (MDA) of **a**ntibiotics, promotion of **f**acial cleanliness and **e**nvironmental improvement, collectively termed the SAFE strategy. The aim of the A, F and E components of the SAFE strategy is to reduce prevalence and transmission of ocular *Ct* infection. Decisions to start and stop these interventions are based on whether the prevalence of trachomatous inflammation—follicular (TF) is ≥5%.^3^^,^^4^

TF was introduced in the World Health Organization (WHO) simplified trachoma grading scheme to enable assessment of trachoma by non-specialist personnel on a large scale.^5^ TF is often asymptomatic and self-resolving; it is used as a proxy to estimate the population burden of ocular *Ct* infection. In some settings (for example, low-prevalence^6^ or post-treatment^7^ populations) the specificity and positive predictive value of TF for *Ct* infection is low^8^. Also, while TF is conceptually linked to the likelihood of future scarring pathogenesis and potential to develop TT, in longitudinal studies the number or duration of episodes of TF alone is not associated with incidence of conjunctival scarring.^9^, ^10^, ^11^ Therefore, there is a potential role for non-TF markers in the assessment of trachoma.

Data from the Solomon Islands,^12^ Vanuatu^13^ and Papua New Guinea^14^ have shown the epidemiology of trachoma in Melanesia to be unusual in that TT is rare or absent despite a moderate prevalence of TF. In contrast, in Kiribati, both TF and TT exceed the prevalence threshold for trachoma to be considered a public health problem.^15^^,^^16^ Studies of high-TF villages in the Solomon Islands have shown infection prevalence, anti-Pgp3 antibody prevalence and scarring severity to be low despite high TF prevalence.^17^^,^^18^ The Pacific Islands are, therefore, a setting where deploying additional metrics of *Ct* infection and pathogenesis may be valuable to policy makers as they decide how best to manage trachoma. Here, the measurement of three markers (ocular *Ct* infection, anti-Pgp3 antibodies and conjunctival scarring) was integrated with estimation of population-based prevalence of TF. The aims of this study were to demonstrate the utility of non-TF markers by contrasting the relationship between TF and ocular *Ct* infection in Vanuatu, where infection is suspected to be rare, to that in Kiribati, a neighbouring country where infection is suspected to be more common.

## Materials and methods

### Study ethics and consent procedure

The study was approved by the London School of Hygiene & Tropical Medicine (LSHTM; 11158), the Vanuatu National Ethics Review Board (MOH/DG 01/21 GKT-lr) and the Kiribati Ministry of Health and Medical Services (25/05/16). The study was described verbally to all participants in local languages prior to enrolment. All participants provided written consent to take part in the study. For those under the age of 18 years, a parent or guardian provided written consent on their behalf. Those with clinical signs of trachoma were treated in accordance with regional guidelines.

### Study design

Population-based prevalence surveys were undertaken in two evaluation units: the island of Tarawa in Kiribati and the country of Vanuatu (with urban centres of Port Vila and Luganville excluded). The sample was designed to meet contemporary international standards for pre-MDA trachoma mapping. The design of the study, including sample size target, cluster selection and household selection methodologies were matched to those of the Global Trachoma Mapping Project (GTMP),^19^ therefore we aimed to enrol 1018 children aged 1–9 years per EU. The number of households per cluster was chosen based on the number that could be feasibly examined in one day (25 in Tarawa, 30 in Vanuatu) and the number of clusters was determined by the number that would yield the appropriate sample size, based on the expected number of children per household in recent census data (28 clusters in Tarawa, 36 in Vanuatu).

The survey in Tarawa took place in August–September 2016. The survey in Vanuatu took place in June–July 2016. Both surveys were completed prior to any nationwide intervention for trachoma. In Vanuatu, mass drug administration of azithromycin (at a dose of 30 mg/kg to a maximum of 2 g) had taken place in Tafea Province, the southern part of Malekula island and the southern part of Espiritu Santo island in 2012 to control yaws.

### Clinical examination

Nurses with experience in grading trachoma were re-trained to evert eyelids, to collect swabs and to grade three key disease signs from the WHO simplified grading system: TF, TI and TT.^5^ Graders were certified prior to survey fieldwork using the GTMP grader training scheme.^19^ Data were recorded on smartphones using Open Data Kit (ODK; odk.lshtm.ac.uk) and sent to servers at LSHTM for cleaning and curation. TF prevalence estimates were adjusted for age and gender in one-year age categories, TT prevalence estimates were adjusted for age and gender in five-year age categories according to most recent census data.^20^^,^^21^

### Conjunctival scarring

Following clinical examination of the right eye, a digital photograph was taken of the conjunctiva. Photographs were linked to participant information and then reviewed (masked to demographic details) by two independent photograders. Photographs were graded for conjunctival scarring according to the 1981 WHO grading scheme.^22^ Photographs were graded as C0–C3 using the following criteria:

• C0: No scarring on the conjunctiva.
• C1: Mild: Fine, scattered scars on the upper tarsal conjunctiva or scars on other parts of the conjunctiva.
• C2: Moderate: more severe scarring, but without shortening or distortion of the upper tarsus.
• C3: Severe: Scarring with distortion of the upper tarsus.

Where the two independent grades agreed, that grade was considered final. Where the primary photograders did not agree, photographs were re-graded by a third, highly experienced photograder and their grade was considered final.

### Ocular *Chlamydia trachomatis* infection

After photography, a sterile, polyester-coated cotton swab was passed three times, with a 120-degree rotation between each pass, over the everted right conjunctiva and placed into a dry, sterile cryotube.^23^ Examiners cleaned their hands with alcohol gel and changed gloves between examinations and took care to avoid cross contamination of specimens. One swab per village was collected to control for cross contamination of swabs in the field (referred to as field controls). Field control swabs were passed within 20 cm of the eye of a seated participant and then treated identically to swabs taken from the conjunctivae of study participants. Swabs were stored at ambient temperature for up to 24 h, refrigerated for up to one week and frozen thereafter. Swabs collected in Vanuatu were shipped to the UK on dry ice, whereas the facility to ship on dry ice was not available in Tarawa; swabs collected in Kiribati were therefore shipped with ice packs to the UK. Both swab sets were stored at −20 °C on arrival in the UK.

Swabs were processed between September 2017 and January 2018. DNA was extracted from the swabs using the QIAamp DNA mini kit and eluted in 100 µL TE. A single known-negative specimen was extracted alongside each batch of clinical specimens to test for contamination in the extraction process (referred to as extraction controls). Eluate was tested for presence of host DNA (endogenous control target) and *Ct* plasmid DNA (diagnostic target) using a validated droplet digital PCR assay^24^ with minor protocol modifications as described elsewhere.^17^^,^^25^ Known-positive and no-template controls were run on each plate to check for contamination of reagents and provide guidance for manual threshold gating (referred to as PCR controls).

### Anti-Pgp3 antibodies

Each participant had their finger cleaned and pricked, and a few drops of blood collected onto extensions of a filter paper disk estimated to hold 10 µL of blood. Blood spots were dried at ambient temperature overnight, then refrigerated for up to one week before freezing.^26^ Dried blood spots (DBS) were shipped to the UK at room temperature and re-frozen on arrival then processed in January–June 2018. Anti-Pgp3 antibody level was measured with an ELISA assay, as described elsewhere.^27^^,^^28^ A blocking step was added to the published protocol for this study: after antigen coating, plates were incubated for one hour at room temperature on a rocking platform with 100 µL phosphate-buffered saline with 0.3% Tween-20 and 5% weight-for-volume milk powder. Optical density (OD) was normalised as previously described.^27^ Thresholds for positivity were determined as previously described.^29^ As there are genetic differences between the majority ethnicities in Tarawa and Vanuatu,^30^ thresholds for samples from the two EUs were set independently (normalised OD of 0.568 and 0.703, respectively).

### Data analysis

Seroconversion rates (SCRs) in children aged 1–9 years were estimated from age-specific seroprevalence curves using a simple reversible catalytic model (RCM) as described elsewhere, using parameters as recommended by those authors.^31^ Based on the epidemiological context, we used a RCM in which the SCR was assumed to be constant for all individuals aged between 1 and 9 years because of the lack of recent interventions against trachoma. Because of the low seroprevalence, the seroreversion rate (SRR) was assumed to be 0 events per person per year, as demonstrated for seroepidemiological studies of malaria.^32^ The model deployed corresponded to scenario 1 version 2 of the scenarios tested by Pinsent et al.^31^

Proportions were compared using Fisher's Exact Test. For small, non-parametric group comparisons (such as comparing the load of infection in those with or without TF in Vanuatu), Mann Whitney U tests were used. Associations between binary dependant variables were tested by logistic regression with age and gender added as *a priori* independent variables.

## Results

### Enrolment and prevalence of signs of disease

**Fig. 1:**
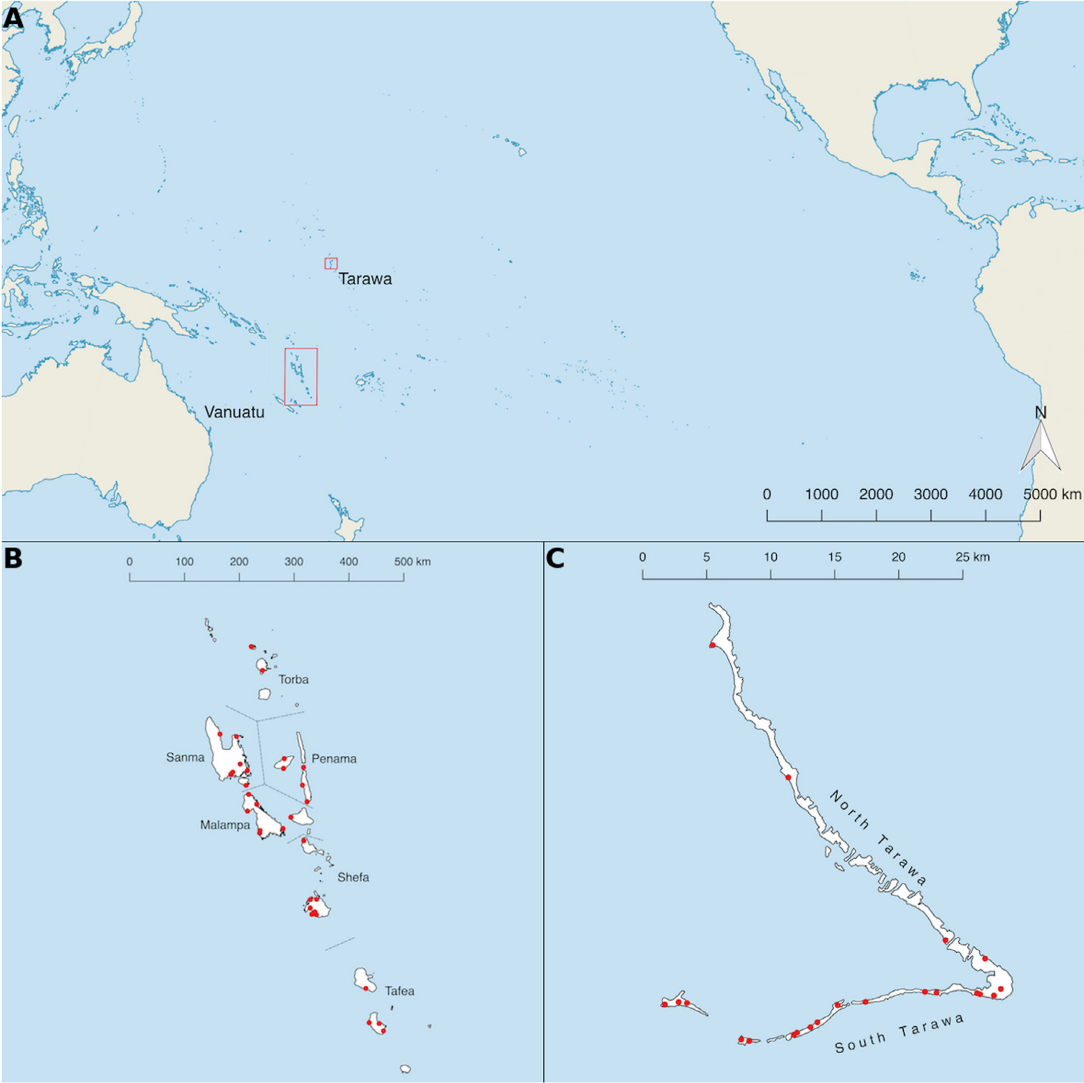
(**A**) Location of evaluation units surveyed in 2016. (**B**) Cluster locations (red dots) in Vanuatu. (**C**) Cluster locations (red dots) in Tarawa, Kiribati. Shapefiles obtained from Database of Global Administrative Areas (www.gadm.org).

Cluster locations are shown in Fig. 1. Fewer clusters were visited than originally planned because the sample size was reached early. In Vanuatu 3470 people ≥1 year were recruited from 33 clusters, and in Tarawa 2922 people ≥1 year were recruited from 22 clusters. According to the most recent census data, the median age of the target age group (≥1 years) in Vanuatu was 21 years (inter-quartile range [IQR]: 10–37 years) and the population was 52% male. The median age in our sample from Vanuatu was 20 years (IQR: 7–39 years) and 47% were male. In Kiribati, the most recent census suggested the median age in the target population was 22 years (IQR: 11–39) and 51% were male. The median age in our sample from Kiribati was 19 years (IQR: 6–38 years) and 40% were male. The age and gender distributions of sampled individuals in the two EUs were, therefore, similar to each other. Adult males (the lowest risk group for trachoma and trachoma-associated pathology) were under-represented in both surveys. The prevalence of TF, TI and TT is shown in Table 1. The peak age-specific TF prevalence was in those aged 13 years (63%) in Tarawa and in those aged 10 years (21%) in Vanuatu.

**Table 1:** Prevalence of selected signs of trachoma in Vanuatu and Tarawa, Kiribati, 2016.

EU (population estimate*)	Age group (years)	Number examined	% male	TF	TI ± TF	Unadjusted* TF prevalence (95% CI)	Adjusted* TF prevalence (95% CI)	TT (%)	Unadjusted TT prevalence (95% CI)	Adjusted* TT prevalence (95% CI)
Vanuatu (176,828)	1–9	1112	52.5	184	0	16.5 (14.3–18.7)	16.5 (14.3–18.7)	–	–	–
	10–14	418	51.9	60	0	–	–	–	–	–
	≥15	1940	42.1	15	0	–	–	0	0 (0.0–0.2)	0 (0.0–0.3)
Tarawa (63,017)	1–9	1059	49.5	436	7	41.1 (38.1–44.1)	38.2 (35.7–41.5)	–	–	–
	10–14	252	53.6	141	6	–	–	–	–	–
	≥15	1611	31.8	119	30	–	–	18	1.1 (0.6–1.6)	0.8 (0.4–1.2)

⁎ Population estimates and age/gender adjustments calculated using most recent census data in Vanuatu (20) and Kiribati (21). TF estimates were adjusted for age and gender in one-year age bands and TT estimates were adjusted for age and gender in five-year age bands. CI, confidence interval; EU, evaluation unit; TF, trachomatous inflammation—follicular; TI, trachomatous inflammation—intense; TT, trachomatous trichiasis.

### Conjunctival scarring

**Fig. 2:**
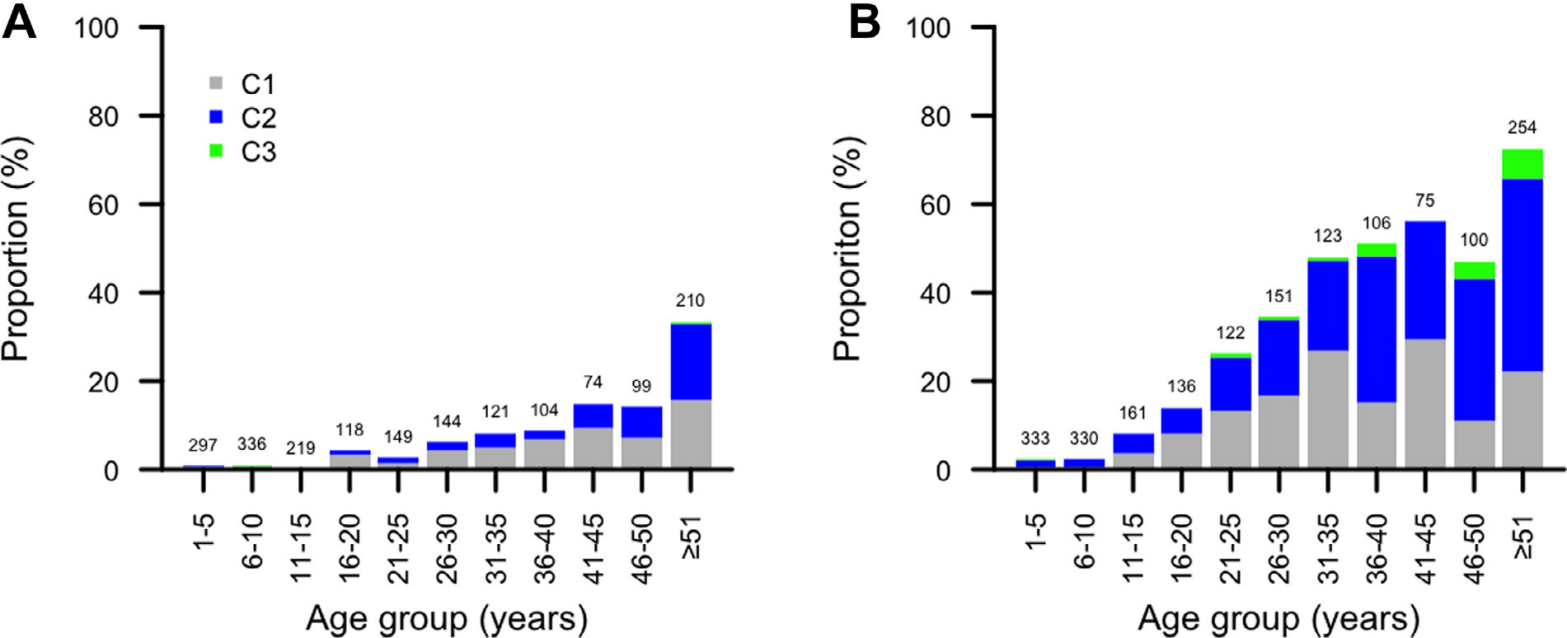
Age-specific prevalence of conjunctival scar grade in (**A**) Vanuatu (*n* = 1871) and (**B**) Tarawa, Kiribati (*n* = 1891). Group sizes are displayed above each bar. C0: No scarring on the conjunctiva (not shown); C1: Mild: Fine, scattered scars on the upper tarsal conjunctiva or scars on other parts of the conjunctiva; C2: Moderate: more severe scarring, but without shortening or distortion of the upper tarsus; C3: Severe: Scarring with distortion of the upper tarsus.^22^

In Vanuatu and Tarawa, 1871/3470 (54%) and 1891/2922 (65%) participants had gradable photographs. The age-specific prevalence of conjunctival scarring is shown in Fig. 2. A larger proportion of photographs from Kiribati had evidence of scarring of any severity than in Vanuatu (27% versus 7%, Fisher's Exact test, *p* < 0.0001), and in older people the difference was similarly marked; the proportion of those aged ≥51 years with any scar was 33% in Vanuatu and 72% in Tarawa (Fisher's exact test *p* < 0.0001). A multivariable regression model was run with any scar as the dependant variable and age (as a continuous variable), gender, an EU as independent variables. In that comparison, scars were more common in older people (odds ratio [OR]: 1.07, 95% confidence interval [CI]: 1.06–1.08; *p* < 0.001), more common in females than males (OR: 1.4, 95% CI: 1.2–1.8; *p* = 0.001) and less common in Vanuatu than Tarawa (OR: 0.13, 95% CI: 0.10–0.16; *p* < 0.001).

### Ocular *Chlamydia trachomatis* infection

All field control swabs tested negative for both *Ct* and human DNA targets. In Vanuatu, 1040/1112 (94%) children aged 1–9 years had swabs which were positive for the endogenous control target; however, the extraction controls processed alongside 141 samples suggested reagent contamination during extraction, and these samples were removed from the analysis. Therefore, results for 899 children were included. Of these, 16/899 (1.8%) had evidence of *Ct* infection. The age- and gender-adjusted prevalence of infection was 1.5% (95% confidence interval [CI]: 0.8–2.2%). There was no association between ocular *Ct* infection and active trachoma (logistic regression adjusted for age and gender *p* = 0.71, odds ratio [OR]: 1.3 [95% CI: 0.3–4.0]). The median load of infection in those with *Ct* infection was low (96 plasmid copies/swab, range: 66–8160 plasmid copies/swab).

In Tarawa, 1009/1059 (95%) of children aged 1–9 years had swabs which were positive for the endogenous control target. Of these, 293 (29.0%) had evidence of *Ct* plasmid DNA. When adjusted for age and gender in one-year bands, the adjusted prevalence of infection was 27.4% (95% CI: 24.7–30.1%). Prevalence of ocular *Ct* infection was higher in children with active trachoma (logistic regression adjusted for age and gender *p* < 0.0001, OR: 4.3 [95% CI: 3.2–5.8]; Table 2). The median load of detected *Ct* infections was high (16,758 plasmid copies/swab, range: 54–5,000,000 plasmid copies/swab). Amongst children with positive swabs, the load of infection was higher in those with active trachoma in the right eye (the eye from which swabs were taken) than in those without right eye active trachoma (linear regression *p* = 0.0001).

**Table 2:** Relationship between active trachoma and ocular *Ct* infection in Vanuatu and Tarawa, Kiribati.

Evaluation unit	ddPCR (%)	TF ± TI in the right eye		
		No	Yes	Total
Vanuatu	Negative	731 (98.3)	152 (98.1)	883 (98.2)
	Positive	13 (1.7)	3 (1.9)	16 (1.8)
	Total	744	155	899
Tarawa	Negative	521 (83.0)	195 (51.2)	716 (71.0)
	Positive	107 (17.0)	186 (48.8)	293 (29.0)
	Total	628	381	1009

### Anti-Pgp3 antibodies

In Vanuatu, 3401/3470 (98%) DBS were tested for anti-Pgp3 antibodies, of which 1084 were from children aged 1–9 years. In Tarawa, 2805/2922 (96%) DBS were tested for anti-Pgp3 antibodies, of which 1015 were from children aged 1–9 years. We did not collect data on the reasons for non-collection of DBS from the remaining individuals studied. In Vanuatu, the anti-Pgp3 seroprevalence in those aged 1–9 years was 88/1084 (8%) but increased rapidly between the ages of 16–25 years. The anti-Pgp3 seroprevalence in those aged ≥18 years was 50%. In Tarawa, the anti-Pgp3 seroprevalence in those aged 1–9 years was 600/1015 (59%) and increased rapidly throughout the childhood years. The anti-Pgp3 seroprevalence in those aged ≥18 years was 90%. Antibody data were available from 14 cases of infection in Vanuatu and 290 infection cases in Kiribati. In Vanuatu, 3 out of 14 (21%; 95% CI: 6.7–57.2%) were seropositive. In Kiribati, 235 out of 290 (81%; 95% CI: 75.9–85.3%) were seropositive

**Fig. 3:**
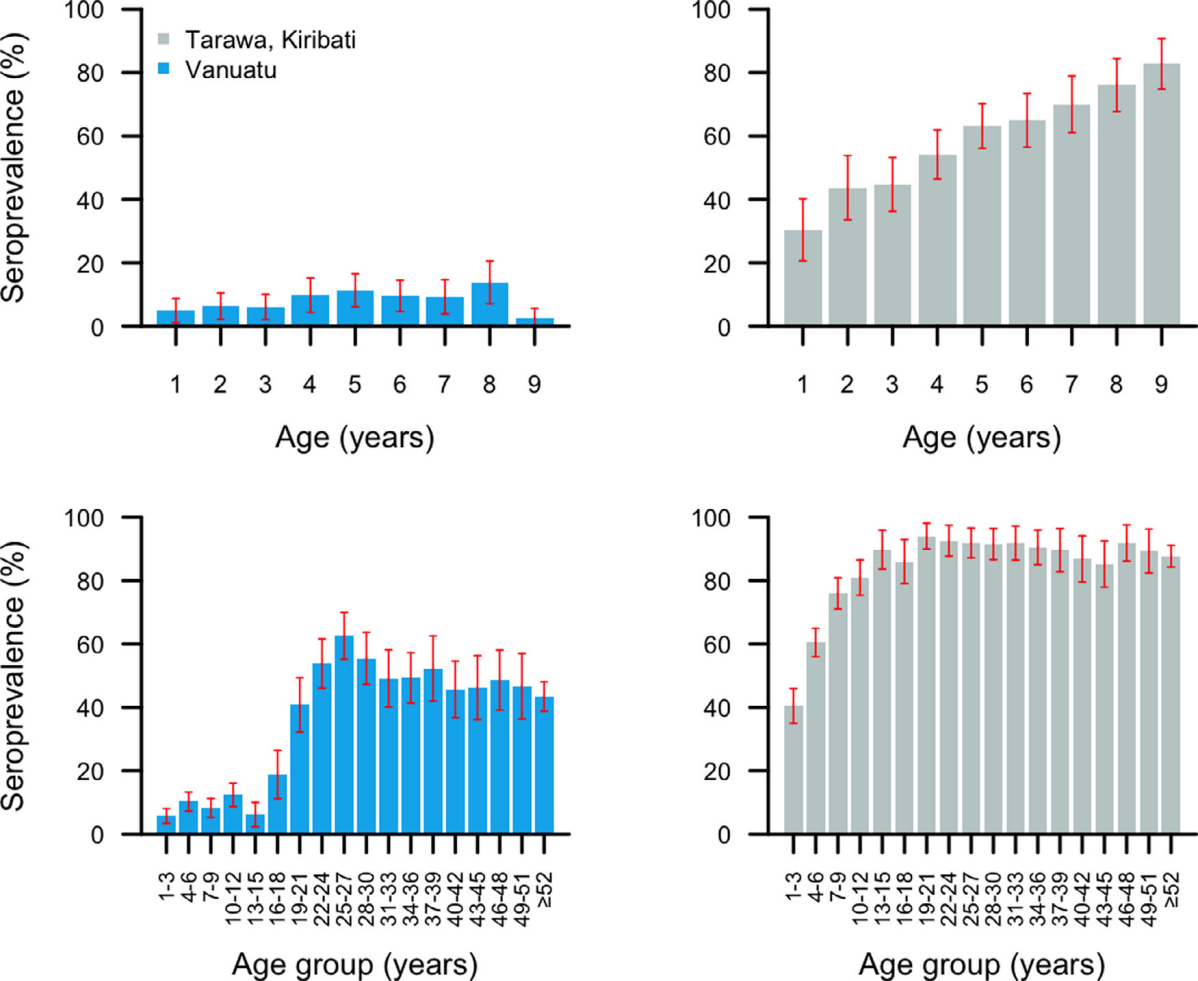
Upper panels show age-specific anti-Pgp3 seroprevalence in children aged 1–9 years in (**A**) Vanuatu (*n* = 1084) and (**B**) Tarawa, Kiribati (*n* = 1015). Lower panels show age-specific anti-Pgp3 seroprevalence in people of all ages in (**C**) Vanuatu (*n* = 3401) and (**D**) Tarawa, Kiribati (*n* = 2805). Samples collected June–September 2016. Red whiskers indicate 95% confidence intervals.

From the RCM, the SCR in Vanuatu was estimated at 0.018 (95% credible interval [CrI]: 0.014–0.022) seroconversion events per 1–9-year-old per year. In Tarawa, the SCR in those aged 1–9 years was 0.197 (95% CrI: 0.181–0.214) seroconversion events per 1–9-year-old per year. The mean difference between parameter estimates for the two study sites is 0.180 (95% CrI: 0.162–0.197); as the credible interval does not include zero, we would consider these to be significantly different. The age-specific seroprevalence in each EU is shown in Fig. 3. In Vanuatu, there was no association between active trachoma in either eye and anti-Pgp3 antibody level, whereas in Tarawa anti-Pgp3 antibody levels were significantly higher in children with active trachoma (logistic regression adjusted for age and gender *p* = 0.93 and *p* < 0.0001, respectively).

## Discussion

As the trachoma elimination programme continues and emphasis shifts towards peri-elimination surveys and post-elimination surveillance, more specific diagnostics may be required to evaluate need for interventions. In this study, two trachoma-endemic populations were surveyed using a range of potential tools for monitoring trachoma and assessing the need for MDA.

The clinical data collected in both locations of this study were broadly similar to the pre-treatment data generated by the respective national programmes. All pre-treatment studies (those presented here and those completed separately by the national programmes) followed a similar study design, had similar sample sizes and had similar age and gender profiles. There were, however, significant differences in the TF prevalence estimates in both study sites. In Vanuatu, the national programme's pre-MDA TF prevalence estimate was 12% (95% CI: 8.1–16.7%) in October–November 2014,^13^ whereas in this survey, carried out in June–July 2016, it was higher at 16.5% (95% CI: 14.3–18.7%). In South Tarawa, the national programme's pre-MDA TF prevalence estimate was 21.3% in 2012.^15^ In our August–September 2016 survey of South and North Tarawa, the prevalence was higher at 38.2%. This could suggest sustained increases in TF prevalence in both areas, seasonal variation in TF prevalence or artefact due to sampling variation. Both the current estimate and the programme's previous estimate of TT prevalence in the ≥15-year-old age group in Vanuatu suggest TT to be below the target threshold for elimination as a public health problem. In Tarawa, both estimates of TT prevalence were above the target threshold. Therefore, according to clinical signs alone, implementation of the A, F and E components of the SAFE strategy is warranted in both populations and the S component is needed in Tarawa.

The scarring and infection data collected provide a more detailed impression of trachoma in each EU. Conjunctival scarring was more prevalent in Tarawa than in Vanuatu, which probably explains the difference in TT prevalence between the two locations. Compared to previous population-based estimates of scar prevalence and severity, Tarawa is similar to areas with high ocular *Ct* infection prevalence,^33^ and Vanuatu is similar to areas in Melanesia with low ocular *Ct* infection prevalence.^18^

Ocular *Ct* infection was common in Tarawa, with almost 30% of the children aged 1–9 years having infection. The relationship between TF and ocular *Ct* infection in Tarawa resembles trachoma-endemic districts in sub-Saharan Africa.^34^ In contrast, the relationship between ocular *Ct* infection and TF in Vanuatu resembles findings from neighbouring Solomon Islands and Papua New Guinea,^17^^,^^35^ with the ocular *Ct* infection prevalence being considerably lower than the prevalence of TF. Notably, the absence of association between TF and anti-Pgp3 antibody in Vanuatu suggests that TF there may have an alternative, non-chlamydial cause. Data from the Solomon Islands investigating this pattern suggest that the cause may not be bacterial.^36^ Another marker of interest for assessing severity of disease is intense inflammation. Intense inflammation is consistently demonstrated to be associated with incidence and progression of scarring in longitudinal studies and prevalence and load of *Ct* infection are higher in people with TI than in those with TF. In this study, the TI prevalence did not differ between 1 and 9-year-olds in Kiribati and in Vanuatu.

The age-specific anti-Pgp3 seroprevalence curve in Tarawa suggests a cumulative exposure to *Ct* over childhood years, culminating in near-universal exposure by early adolescence. This has been observed in a number of infection-endemic settings^37^ including elsewhere in Kiribati.^16^ The SCR suggests *Ct* transmission is intense in Tarawa. Conversely, in Vanuatu the low anti-Pgp3 seroprevalence and SCR in children aged 1–9 years suggests overall exposure to and transmission of *Ct* in this age group are low. Steep increases in age-specific seroprevalence rates are observed in late adolescence and early adulthood. This coincides with the median age of sexual debut in Vanuatu^38^ and is likely to be attributable to urogenital *Ct* exposure. Comparing the age-specific seroprevalence profiles from Vanuatu to those from the Solomon Islands shows that the mean seroprevalence in 1–9-year-olds and in adults is higher in the Solomon Islands; it is interesting to note that, according to some recent estimates, the urogenital *Ct* infection rate is also higher in the Solomon Islands than in Vanuatu.^39^^,^^40^

Overall, the prevalence of infection and scaring in Tarawa support the clinical data in justifying antibiotic MDA for trachoma. In Vanuatu, the scarring and infection data suggest that the absence of TT may be linked to the relative absence of *Ct* transmission, similar to what has been suggested in the Solomon Islands. Therefore, it seems logical that the Melanesian experience of trachoma is different to that seen in Kiribati and sub-Saharan Africa.

There are limitations to this work. While the sample size to estimate TF prevalence was achieved in both EUs, there were missing data for some of our analyses. We did not collect data on the non-response rate in the survey in either EU. A small proportion of individuals were examined clinically but then did not have either or both of the swab or DBS collected. The reasons for this were not systematically recorded, but field workers anecdotally reported that if the examinations were running slowly or were uncomfortable to those examined, participants were more likely to refuse specimen collection. For the scarring analysis, a significant proportion of people in the study did not have a gradable photograph. The use of photographs has previously been found to be less than optimal for grading conjunctival signs of trachoma.^41^ Should future studies utilise clinical photography to diagnose conjunctival scarring, additional field-based checks should be put in place to ensure image quality at the time of capture. For the SCR estimation, the models used for estimation of SCRs were simplified to exclude parameters which are not yet fully understood. For example, we did not account for the influence of non-ocular *Ct* infection, which is known to be prevalent in both areas. We acknowledge this may reduce the precision of our SCR estimates, however, we elected to maintain simplistic models rather than incorporating parameters which have not been empirically proven. Finally, we also did not collect data on household-level risk factors for ocular *Ct* infection, antibody responsiveness or scarring. Data relevant to potential risk factors for trachoma are available from broadly overlapping EUs surveyed by the national programme (Vanuatu^13^ and South Tarawa^15^). There are socio-cultural and geographical differences between Vanuatu and Tarawa that may be of interest should the differences in infection and scarring profiles be investigated in more detail.

There is a definite need for intervention against trachoma in Tarawa. However, in Vanuatu and other Melanesian countries the need for intervention is less clear. The non-TF markers used in this study help to understand the picture of trachoma in Melanesia in comparison to the neighbouring country of Kiribati, and support suggestions that non-TF markers should be utilised for decision making in Papua New Guinea, Solomon Islands and Vanuatu.^42^ Serological surveillance in particular has clear potential for integration with other disease serosurveillance programmes.^43^ Our data on these additional non-TF markers in this context should stimulate further discussion about their wider role in trachoma surveillance.

## CRediT authorship contribution statement

**Robert Butcher:** Conceptualization, Data curation, Formal analysis, Funding acquisition, Investigation, Methodology, Project administration, Supervision, Validation, Visualization, Writing - original draft, Writing - review & editing. **Becca Handley:** Formal analysis, Investigation, Writing - review & editing. **Mackline Garae:** Investigation, Project administration, Writing - review & editing. **Raebwebwe Taoaba:** Investigation, Methodology, Project administration, Supervision, Writing - review & editing. **Harry Pickering:** Formal analysis, Investigation, Methodology, Writing - review & editing. **Annie Bong:** Investigation, Methodology, Project administration, Supervision, Writing - review & editing. **Oliver Sokana:** Investigation, Supervision, Writing - review & editing. **Matthew J Burton:** Investigation, Validation, Writing - review & editing. **Nuno Sepúlveda:** Formal analysis, Methodology, Writing - review & editing. **Ana Cama:** Conceptualization, Investigation, Methodology, Project administration, Supervision, Writing - review & editing. **Richard Le Mesurier:** Conceptualization, Funding acquisition, Methodology, Writing - review & editing. **Anthony W. Solomon:** Conceptualization, Funding acquisition, Methodology, Supervision, Writing - review & editing. **David Mabey:** Conceptualization, Funding acquisition, Methodology, Supervision, Writing - review & editing. **Fasihah Taleo:** Conceptualization, Funding acquisition, Investigation, Methodology, Supervision, Writing - review & editing. **Rabebe Tekeraoi:** Conceptualization, Funding acquisition, Investigation, Methodology, Supervision, Writing - review & editing. **Chrissy h Roberts:** Conceptualization, Funding acquisition, Methodology, Supervision, Writing - review & editing.

## Declaration of Competing Interest

None of the authors declare any conflicts of interest.
